# Analysis of CO_2_ Absorption in Gas/Liquid Membrane Contactors with Inserted Descending Hydraulic Diameters of 3D-Printed Turbulence Promoters

**DOI:** 10.3390/membranes15030088

**Published:** 2025-03-09

**Authors:** Chii-Dong Ho, Yi-Wun Wang, Zheng-Zhong Chen, Thiam Leng Chew

**Affiliations:** 1 Department of Chemical and Materials Engineering, Tamkang University, Tamsui, New Taipei 251301, Taiwan; ywwangi@mail.tku.edu.tw (Y.-W.W.); 611400598@o365.tku.edu.tw (Z.-Z.C.); 2 Department of Chemical Engineering, Faculty of Engineering, Universiti Teknologi PETRONAS, Seri Iskandar 32610, Perak, Malaysia; thiamleng.chew@utp.edu.my; 3 Centre of Carbon Capture, Utilization and Storage (CCCUS), Institute of Sustainable Energy & Resources (ISER), Universiti Teknologi PETRONAS, Seri Iskandar 32610, Perak, Malaysia

**Keywords:** CO_2_ absorption, sherwood number, flat-plate membrane contactor, concentration polarization, hydraulic diameters

## Abstract

The decline in absorption flux across membrane modules is attributed to the increase in concentration polarization resistance in flat-plate membrane contactors for CO_2_ absorption using monoethanolamine (MEA) as the absorbent. Researchers have discovered that this effect can be mitigated by inserting turbulence promoters, which enhance turbulence intensity at the cost of increased power consumption, thereby improving CO_2_ absorption flux. The performance of flat-plate membrane contactors for CO_2_ absorption was further enhanced by reducing the hydraulic diameters of embedded 3D-printed turbulence promoters, considering the increased power consumption. The mass-balance modeling, incorporating chemical reactions, was developed theoretically and conducted experimentally on a flat-plate gas/liquid polytetrafluoroethylene/polypropylene (PTFE/PP) membrane module in the present study. A one-dimensional theoretical analysis, based on the resistance-in-series model and the plug-flow model, was conducted to predict absorption flux and concentration distributions. An economic analysis was also performed on modules with promoter-filled channels, considering different array configurations and geometric shapes of turbulence promoters, weighing both absorption flux improvement and power consumption increment. Device performances were evaluated and compared with those of modules using uniform promoter widths. Additionally, the Sherwood number for the CO_2_ membrane absorption module was generalized into a simplified expression to predict the mass transfer coefficient for modules with inserted 3D-printed turbulence promoters. Results showed that the ratio of absorption flux improvement to power consumption increment in descending hydraulic-diameter operations is higher than in uniform hydraulic-diameter operations.

## 1. Introduction

Membrane absorption is a promising alternative technology with high absorption efficiency for removing undesirable gases such as CO_2_ and H_2_S to reduce greenhouse gas emissions [1]. This technology is widely applied in industrial processes and offers benefits such as low energy consumption, a large mass-transfer area, continuous operation, and scalability [2]. Flue gases from fossil fuel combustion contain CO_2_, which must be removed to address environmental concerns related to global warming [3]. Impurities, including CO_2_ (30–45%) and H_2_S (0.5–1%), which are major contributors to the greenhouse effect and climate change [4], are reduced to acceptable levels during biogas upgrading and stabilization processes [5]. This enhances the utility and value of the gas, ensuring it meets the specifications required for biogas processing, conditioning, or pipeline transport. The need for research into and development of low-energy, high-efficiency technologies to capture and remove CO_2_ has been the subject of major studies, focusing on the use of aqueous amine solutions to achieve higher separation efficiency [6]. The applications of membrane technology are the most common techniques for purifying selectively absorbed soluble CO_2_ mixture components, allowing CO_2_ to diffuse into the liquid phase via mass transfer through a microporous membrane in liquid/liquid and gas/liquid systems [7]. Membrane contactors dominate as an emerging and attractive membrane process with high separation efficiency for CO_2_ capture [8], particularly in addressing greenhouse gas emissions [9] from fossil fuel combustion. Membrane contactor technology enables the liquid absorbent and gas (flue gas) to come into contact, providing a large effective contact surface area for mass transfer in continuous operations, resulting in superior CO_2_ capture efficiency performance [10]. The limitations of conventional contactors can be effectively addressed by implementing membrane contactors, which combine the techniques of traditional gas absorption and membrane separation [8]. This efficiency is attributed to the chemical potential between the two sides of the hydrophobic microporous membrane, where the membrane pores at the gas/liquid interface separate specific substances [11]. This technology also addresses the shortcomings of conventional packed-column absorption processes, such as entrainment, flooding, channeling, and foaming [12]. Membrane contactors provide several advantages for industrial-scale CO_2_ capture processes, including low energy consumption, a large and stable gas/liquid contact area, continuous operation, high modularity, and flexible scalability [2]. The efficiency of separating specific substances depends on the distribution coefficient and the concentration gradient of the gas solute in the gas/liquid system [13]. The operational gauge pressure of both the gas and liquid phases was measured for the membrane contactors [14]. Additionally, the temperature and pressure dependence of CO_2_ absorption through ceramic hollow fiber membrane contactor (CHFMC) modules was evaluated during the experimental runs [15]. The differential pressure across the membrane modules was manually regulated on the liquid side using a needle valve [16]. Absorption efficiency is based on the solubility of CO_2_ in the solvent, encompassing both physical and chemical absorption [17]. Various amines and mixed amines [18] as prospective absorbents [19] have been widely studied for CO_2_ capture efficiency, considering factors such as regeneration cost [20] and capture cost [21]. MEA absorbents have been used to improve the CO_2_ absorption rate in hydrophobic microporous membrane contactor systems [22]. Previous research has examined the mass-transfer mechanisms of CO_2_ absorption in MEA solutions [23] due to their large absorptive capacity and effective absorption, aiming to achieve high-purity products [24]. The dusty gas model [25] has been applied to estimate process performance [26] and mass transfer resistance [27], describing the mass-transfer characteristics across the membrane associated with reactions and diffusion [28].

Membrane contactors suffer from concentration polarization near the membrane surface, leading to decreased mass transfer performance [29]. The impact of concentration polarization is dependent on the feed composition and becomes more pronounced with increasing feed concentration [30]. Turbulence promoters are used to suppress the boundary layer between the bulk and membrane surface formed by the polarization effect, which results in decreased heat and mass transfer rates [31] in membrane separation processes. The resultant turbulence diminishes the polarization effect and increases the mass transfer rate [32], resulting in flux enhancement compared to modules without turbulence promoters. Various types of turbulence promoters include roughened surfaces [33], spacer-filled channels [34], and carbon-fiber spacer channels [35]. This approach has been effectively applied in flat-plate modules, tubular membranes with spiral ring-pitch channels, such as concentric-tube thermal diffusion columns [36,37], and ultrafiltration using wire-rod tubular membrane modules [38]. Spacers and filaments acting as turbulence promoters are commonly employed in membrane contactors to create eddy currents and flow disruption, diminishing the concentration polarization effect and enhancing device performance. An effective strategy for capturing CO_2_ under turbulent flow conditions was investigated by embedding eddy promoters [39] into a parallel-plate gas/liquid membrane contactor. This approach significantly enhanced absorption efficiency compared to modules with a laminar flow velocity profile in the flow channel. Increased turbulence intensity by embedding turbulence promoters in the gas/liquid membrane contactor system has been reported to improve the mass transfer coefficient [40] and to impart additional pressure drop in the feed channel [41]. However, the benefits of using turbulence promoters in membrane separation processes are sometimes outweighed by their disadvantages. This paper examines the theoretical predictions and experimental results of CO_2_ absorption flux using MEA as an absorbent in parallel-plate gas/liquid membrane contactors equipped with 3D-printed turbulence promoters [34]. These promoters were configured with descending hydraulic diameters to examine the effects of increasing shear rate on disturbing the concentration boundary layer, aiming to develop various hydrodynamic conditions with relatively lower power consumption increment.

The implementation of turbulence promoters highlights their hydrodynamic influence on mass-transfer mechanisms, demonstrating technical feasibility and significantly enhancing device performance. The mass-balance modeling, incorporating chemical reactions, was developed theoretically and conducted experimentally on a flat-plate gas/liquid membrane module in the present study. The effects of increased turbulence intensity on the device performance of the absorption process were assessed using a dimensionless quantity called the mass-transfer enhancement factor. Mass-transfer coefficients were analyzed based on the extent of the mass-transfer enhancement factor, which was related to the augmented mass-transfer coefficients and expressed in terms of the correlated Sherwood number. This factor correlates MEA feed concentration and MEA feed flow rates under array configurations of uniform and varying promoter widths. It is believed that the availability of such a simplifying mathematical formulation as developed here for flat-plate membrane modules is valuable in the present work. The same procedure undoubtedly occurs in dealing with many membrane separation processes for further particular applications. This study extends previous work with uniform promoter widths [42] by using descending hydraulic diameters in the MEA feed stream to achieve higher absorption flux and flux improvement. Two geometric shapes of 3D-printed turbulence promoters, say diamond-type and circle-type, were used. The implementation of 3D-printed turbulence promoters [43] in the membrane contactor system was found to increase the pressure drop in the feed channel [41]. Device performances were evaluated and compared with those of modules using uniform promoter widths. It was found that the increased mass transfer resistance due to the insertion of eddy promoters could be mitigated by altering hydrodynamic conditions [44] and appropriately managing the magnitude of energy consumption increment. The trade-off of increased energy consumption [45] was also evaluated to assess the economic viability and technical feasibility of membrane module operations.

## 2. Membrane Absorption Apparatus and Materials

**Figure 1:**
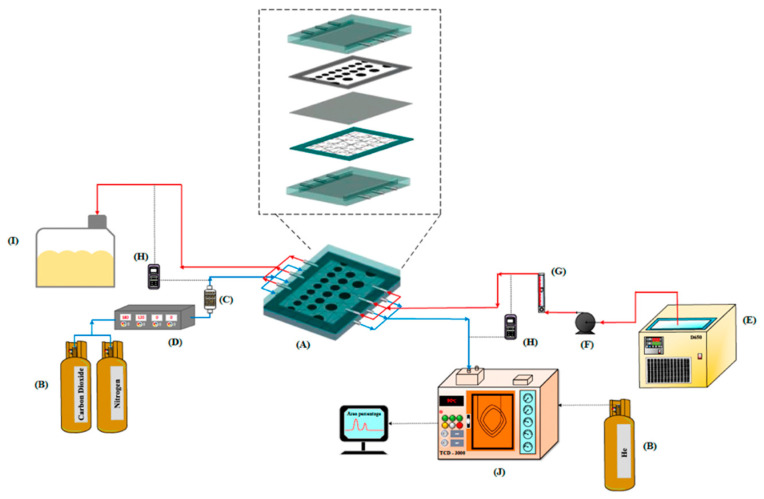
Scheme of a setup used in experiments of flat-plate membrane contactor modules (A—Membrane absorption module; B—Gas cylinder; C—Gas mixing cylinder; D—Mass flow controller; E—Thermostatic tank; F—Pump; G—Flow meter; H—Thermometer; I—MEA Collector; J—Chromatograph).

**Figure 2:**
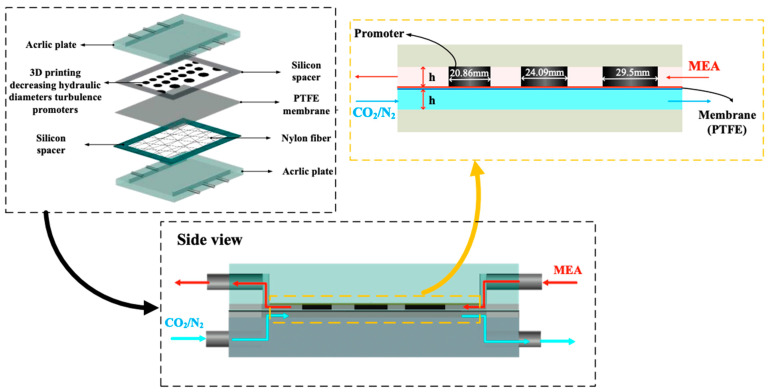
Flat-plate membrane absorption module with 3D printing turbulence promoters.

The schematic configuration and fabrication details of the gas/liquid membrane contactor for CO_2_ absorption using MEA absorbent (Uni-Onward Corp., New Taipei, Taiwan) were conducted in a flat-plate setup and promoter-filled module, as illustrated in Figure 1 and Figure 2, respectively. The experimental setup involves two parallel-plate channels (L = 0.21 m, W = 0.29 m, d = 1 mm) separated by a hydrophobic composite membrane made of PTFE/PP (polytetrafluoroethylene and polypropylene, J020A330R, ADVANTEC, Toyo Roshi Kaisha, Ltd., Tokyo, Japan) with a thermal conductivity of $5.0\times 10^{-4}$ cal/cm sec °C (All-Fluoro Co., Ltd., Taoyuan, Taiwan). The membrane has a nominal pore size of 0.2 µm, a porosity of 0.72, and a thickness of 130 µm (PTFE 98 µm and PP 32 µm), serving as the permeating porous medium in this study. The membrane absorption module contains two flow channels: one with inserted 3D turbulence promoters into the MEA feed flow channel and the other an empty channel for the CO_2_/N_2_ feed stream at 303 K. The empty channel is constructed with a 0.1 mm nylon fiber as a supporting material. The 3D-printed promoters were constructed with a 1 mm thickness using a 3D printer (ATOM 2.5EX, Hsinchu County, Taiwan) and glued with Cyanoacrylate Adhesive (Chang Chun Plastics Co., Ltd., Hsinchu County, Taiwan) onto the acrylic plate of the MEA feed side in contact with the membrane surface, acting as eddy promoters. These promoters were made from polyester elastomer (Polylactic Acid, PLA) with an average molecular weight ranging between 1000 and 60,000 and a density of 1180 kg/m^3^. A 1 mm thick silicone rubber was affixed to the acrylic plate to prevent leakage and to create two spacer conduits of 1 mm for each channel, respectively. This study presents a mathematical modeling of CO_2_ absorption in the MEA absorbent feed channel of the flat-plate membrane module, with a gas mixture of CO_2_/N_2_ flowing through another channel, as depicted in Figure 2.

**Figure 3:**
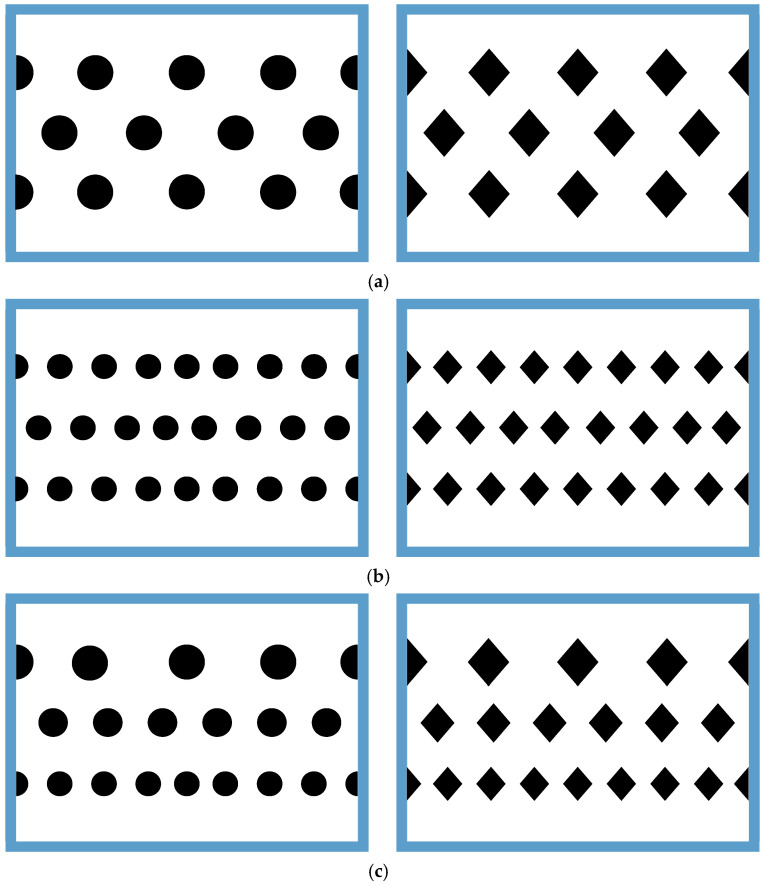
Array configurations of module with the insertion of various 3D-printed turbulence promoters. (**a**) Bigger 3D printing turbulence promoters (circle and diamond types). (**b**) Smaller 3D printing turbulence promoters (circle and diamond types). (**c**) Descending size of turbulence promoters (circle and diamond types).

The 3D-printed promoters of two geometric shapes (circle and diamond types) were fabricated with a 1 mm thickness and inserted into the MEA feed stream. Details of the 3D printing protocol for the experimental work are shown in Figure 3. Figure 3 illustrates the top views of the two geometric shapes of 3D-printed turbulence promoters under various widths and array patterns as design parameters, as specified in Figure 3a–c. The average widths of the circle-type promoters were 18.68 mm, 15.25 mm, and 13.21 mm, respectively, while the average widths of the diamond-type promoters were 16.89 mm, 13.79 mm, and 11.94 mm, respectively. These 3D-printed turbulence promoters provide mechanical strength to prevent membrane vibration and act as eddy promoters. The effective permeate areas were partially hindered by the promoters, which covered approximately 13% of the hydrophobic membrane, a factor accounted for in the calculation procedure. The promoter-filled channel was built by embedding 3D-printed turbulence promoters onto the membrane surface with descending promoter widths, as shown in Figure 3c, while the promoter-filled channel with uniform promoter widths is indicated in Figure 3a,b. The promoters partially obstruct the permeate passages, which reduces gas permeate flux due to the coverage of the membrane surface area and modifies the mass-transfer boundary layers near the membrane surface in the MEA absorbent feed stream.

**Figure 4:**
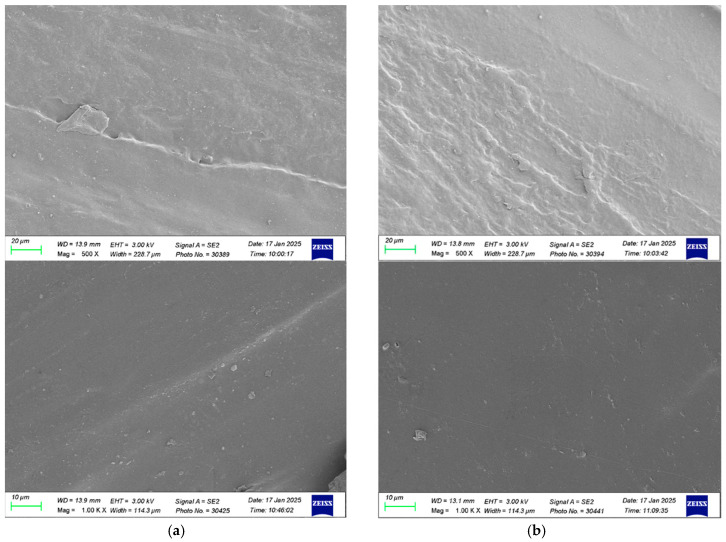
SEM micrographs of the fresh turbulence promoter and used turbulence promoter after experimental runs. (**a**) The fresh turbulence promoter; (**b**) the used turbulence promoter.

Figure 4 shows the SEM (Scanning Electron Microscope, Zeiss Sigma 300, Jena, Germany) micrographs of the fresh turbulence promoter and the used turbulence promoter after experimental runs. The SEM images indicate that the turbulence promoters were effectively manufactured and submerged in the MEA solution for a total durability test time of 48 h. The results demonstrate that the 3D-printed turbulence promoters did not exhibit swelling, confirming their resistance and stability without degradation during the experimental runs.

**Figure 5:**
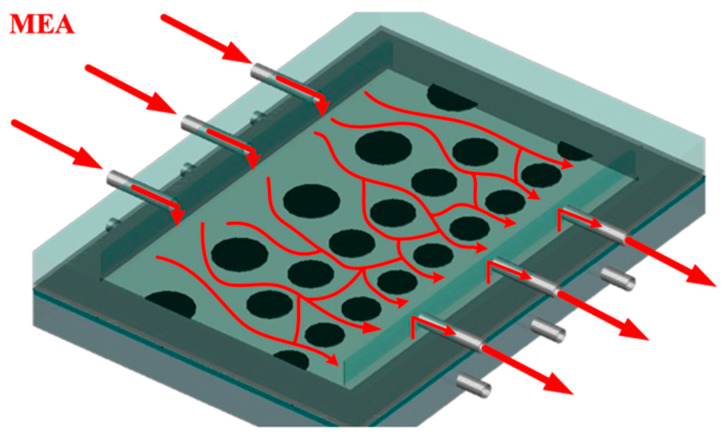
Feed flow streamlines in the descending circle-type widths of 3D-printed turbulence promoters in the MEA absorbent flow channels.

Turbulence intensity augmentation was achieved by implementing 3D-printed turbulence promoters into the MEA absorbent feed channel, acting as eddy promoters with various array configurations and hydraulic diameters. The top view of the geometric circle-type promoters is shown in Figure 5.

**Figure 6:**
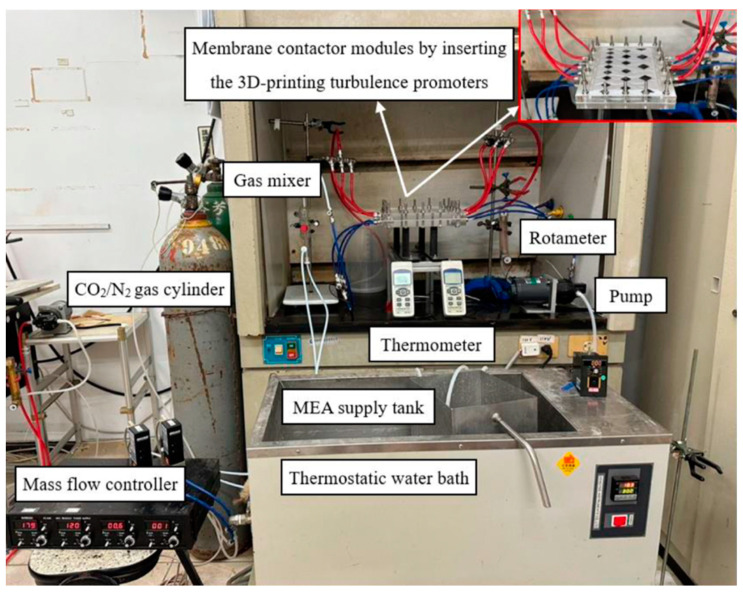
Photographic images of the experimental apparatus for the module with inserted 3D-printed turbulence promoters.

An aqueous MEA absorbent solution was pumped (51K40RA-A, ASTK, New Taipei, Taiwan) from a thermostat (G-50, DENG YNG, New Taipei, Taiwan) to maintain the temperature at 303 K and regulated by a flow meter (MB15GH-4-1, Fong-Jei, New Taipei, Taiwan) to control flow rate through the promoter-filled channel. Various feed flow rates were tested, ranging from 2.5 to 5.0 cm^3^/s (2.5, 3.3, 4.2, 5.0 cm^3^/s). Inlet and outlet temperatures were measured using thermometer probes (TM-946, Lutron, New Taipei, Taiwan) connected to both sides of the flat-plate membrane modules. The MEA absorbent, consisting of 30 wt% MEA, was prepared by diluting MEA with distilled water. A gas mixture containing 30%, 35%, and 40% CO_2_ (balance N_2_) of industrial-grade purity was introduced into the gas mixing tank (EW-06065-02, Cole Parmer Company, IL, USA) and regulated at 5.0 cm^3^/s using a mass flow controller (N12031501PC-540, Protec, Brooks Instrument, Hatfield, PA, USA) to flow into the module until it reached a steady state. The current module was operated with L/G ratios between 500 and 1000 during the experimental runs. These values align with the L/G ratios of 650 to 4800 reported in previous research [46], which also used the same PTFE membrane material. The well-mixed gas feed diffused into and passed through the microporous hydrophobic membrane pores of the membrane absorption contactor. A photo of the operating experimental apparatus of a flat-plate gas membrane absorption system is shown in Figure 6, with acrylic plates as outside walls on a parallel-plate channel.

Comparisons were made of CO_2_ absorption flux under various operating conditions between the flat-plate membrane contactor modules with and without 3D-printed turbulence promoters. All experiments were conducted with the CO_2_/N_2_ gas feed in one-through operations, and the reacted MEA absorbent containing CO_2_ was released into another collector. The CO_2_ exiting the membrane module at a steady state was collected and injected into the column heating systems for rapid heating of the sample-collection capillary tube. The CO_2_ concentrations were standardized and measured using gas chromatography (Model HY 3000, China Chromatograph Co., Ltd., Xinzhuang, New Taipei, Taiwan) with helium as the carrier gas, and conventional thermal conductivity detector (TCD) devices for measurement and recording on a PC. The reproducibility of the CO_2_ concentration measurements was mostly within 5%, allowing for further determination of the CO_2_ absorption flux.

## 3. Theory and Analysis

### 3.1. Mass Transfer

**Figure 7:**
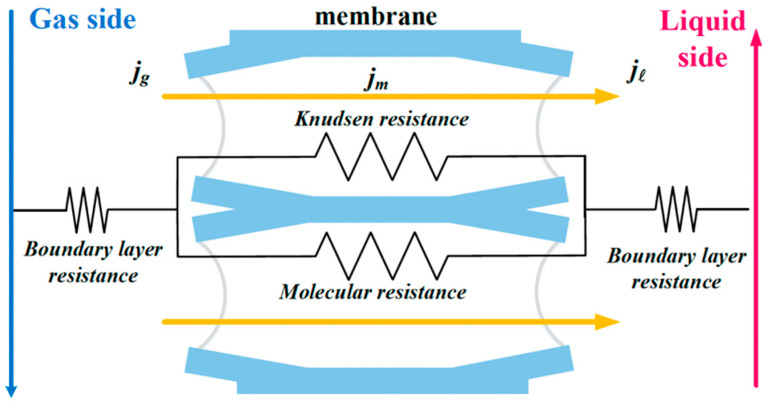
Three regions considered for modeling CO_2_ absorption in a flat-plate membrane absorption module.

**Figure 8:**
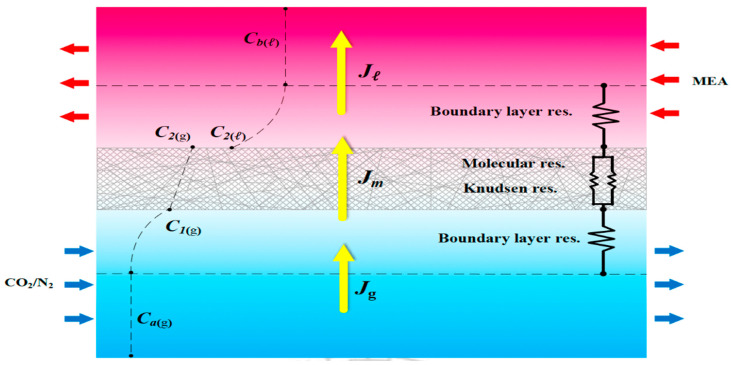
Mass transfer resistances of the CO_2_ absorption flux in a flat-plate membrane contactor module.

The assumptions used in this model include the following: (1) isothermal operation and constant fluid physical properties; (2) the membrane properties are constant; (3) the gas side of the membrane contactor follows ideal gas behavior; (4) the gas phase fills the membrane pores; (5) the membranes are considered to operate in non-wetting mode; (6) the chemical reaction between CO_2_ and MEA takes place only in the liquid phase; (7) Henry’s law is applied at the gas/liquid interface; and (8) the applicability of thermodynamic equilibrium. The process of CO_2_ absorption into an aqueous MEA solution was developed to derive the mathematical model. A representation of the mass-transfer resistance in the membrane contactor is depicted in Figure 7, while Figure 8 graphically presents the bulk concentrations of the gas feed and MEA liquid solution by using the microscopic description, say $C_{a(g)}$ and $C_{b(l)}$, respectively, and both membrane surface concentrations, say $C_{1(g)}$ and $C_{2(g)}$, respectively.

The Henry’s law defined by the dimensionless Henry’s law constant $H_{c}=C_{\left(g\right)}/C_{\left(l\right)}=1.32$ [23] is expressed in terms of the solubility of a gas in a liquid according to the equilibrium of the gas concentration in the liquid phase, or

(1) $$P_{2(g)}=C_{2(g)}RT=hC_{2(l)}$$

or

(2) $$H_{c}=\frac{h}{RT}=\frac{C_{2\left(g\right)}}{C_{2\left(l\right)}}$$

The mathematical modeling equations analyze CO_2_ absorption rates associated with three regions occurring in the isothermal diffusion-reaction processes within the MEA feed channel, as shown in Figure 8. The diffusion-reaction mechanism in the gas/liquid membrane contactor includes three main regions for CO_2_ transferal from the gas mixture feed stream to the MEA absorbent feed stream: (a) the bulk gas diffusion to the membrane surface, (b) transfer through the membrane via its pores, and (c) absorption by the MEA absorbent accompanied by chemical reactions. The mass-transfer coefficients, based on a mass-transfer resistance-in-series model, in gas feed ($k_{a}$), membrane ($K_{m}$), liquid feed ($k_{L}=k_{b}/H_{c}$), and CO_2_ concentration variations are illustrated in Figure 8.

The mass diffusion of CO_2_ was transported by the driving-force concentration gradient through both gas and liquid feed streams, respectively, while the absorption flux $J_{m}$ according to the dusty gas model [25,47] depends on the trans-membrane saturation partial pressure differences (ΔP) [48], as represented below:

(3) $$J_{g}=k_{a}\left(C_{a\left(g\right)}-C_{1\left(g\right)}\right)$$

(4) $$J_{l}=k_{b}\left(K_{ex}^{\prime }H_{c}C_{2(l)}-{H_{c}C}_{b(l)}\right)=k_{L}\left(K_{ex}^{\prime }C_{2(l)}-C_{b(l)}\right),\;k_{L}=k_{b}H_{c}$$

(5) $$J_{m}=c_{m}\left(P_{1}-P_{2}\right)\frac{1}{M_{w}}={\left.c_{m}\frac{dP}{dC}\;\right|}_{C_{mean}}\left(C_{1\left(g\right)}-C_{2\left(g\right)}\right)\frac{1}{M_{w}}\;\;\;\;\;=c_{m}RT(C_{1(g)}-{H_{c}K}_{ex}^{\prime }C_{2\left(l\right)})\frac{1}{M_{w}}=K_{m}(C_{1(g)}-{H_{c}K}_{ex}^{\prime }C_{2\left(l\right)})$$

in which

(6) $$K_{ex}^{\prime }=K_{ex}[\mathrm{MEA}]/[H^{+}],\;K_{ex}=[\mathrm{MEACO}O^{-}]\;[H^{+}]/[CO_{2}][\mathrm{MEA}]=1.25\times 10^{-5}$$

(7) $$c_{m}={\left(\frac{1}{c_{K}}+\frac{1}{c_{M}}\right)}^{-1}={\left\{{\left[1.064\frac{\varepsilon \;r_{p}}{\tau \delta_{m}}{\left(\frac{M_{w}}{RT_{m}}\right)}^{1/2}\right]}^{-1}+{\left[{\left|Y_{m}\right|}_{ln}\frac{D_{m}\varepsilon }{\delta_{m}\tau }\frac{M_{w}}{RT_{m}}\right]}^{-1}\right\}}^{-1}$$

where $K_{m}$ is the overall mass transfer coefficient of the membrane, $K_{ex}^{\prime }$ is the reduced equilibrium constant with the equilibrium constant $K_{ex}$ at T=298 K [49], and the tortuosity τ=1/ε was determined [50]. Meanwhile, the membrane permeation coefficient $c_{m}$ [51] is determined by the membrane properties. Equating the mass transfer fluxes to the conservation law of mass in each region among three intervals, ($C_{a\left(g\right)}-C_{1\left(g\right)})$, $\left(C_{1(g)}-{H_{c}K}_{ex}^{\prime }C_{2\left(l\right)}\right)$ and ($K_{ex}^{\prime }C_{2(l)}-C_{b(l)})$, respectively, as shown in Figure 8, leads to the overall mass transfer coefficient of the gas feed stream and MEA absorbent feed stream, respectively; that is,

(8) $$J_{i}=J_{g}=J_{m}=J_{l},\;i=turbulence\;promoter\;or\;empty\;channels$$

The concentration polarization effect quantifies the degree of membrane mass-transfer resistance, calculated as the ratio of the concentration differences between the membrane surface and the bulk feed stream in both the CO_2_/N_2_ gas mixture and MEA absorbent feed channels. This is referred to as the concentration polarization coefficient.

(9) $$\gamma_{m}=\frac{C_{1\left(g\right)}-{H_{c}K}_{ex}^{\prime }C_{2(l)}}{C_{a\left(g\right)}-H_{c}C_{b\left(l\right)}}$$

**Figure 9:**
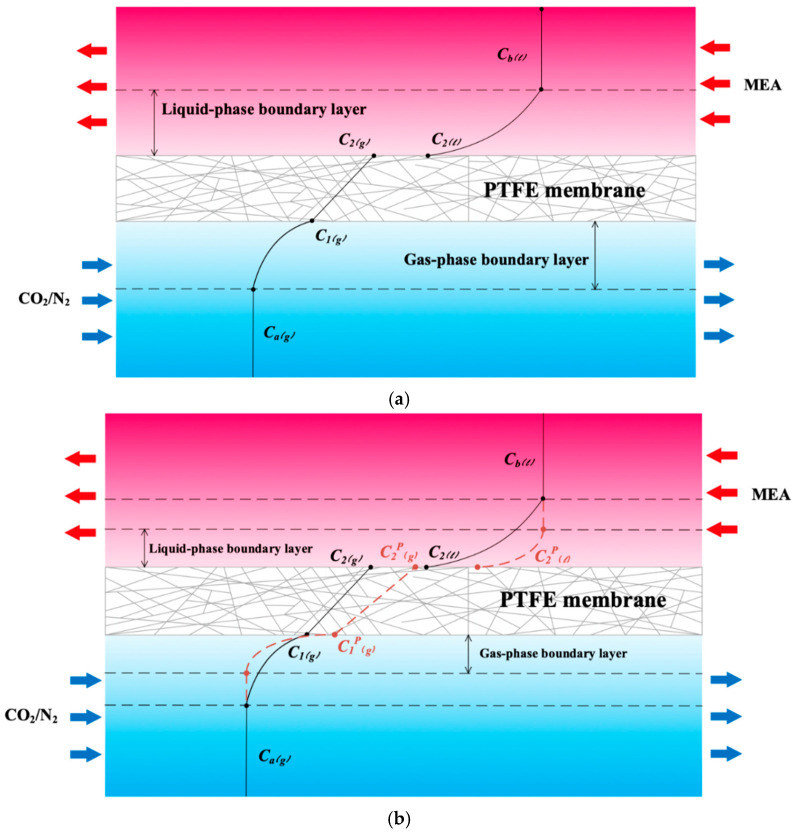
Reduction in mass-transfer boundary polarization layers in the CO_2_ absorption in a flat-plate membrane absorption module. (**a**) Empty channel; (**b**) Channel with inserted 3D-printed turbulence promoters.

Various aspects govern the mass-transfer resistance in membrane absorption modules, and the concentration polarization effect can be reduced by inserting turbulence promoters into the flow channel. Figure 9a,b illustrates an empty channel (without embedding turbulence promoters) and a channel with inserted 3D-printed turbulence promoters. The concentration polarization effect is diminished by using 3D-printed turbulence promoters, as shown in the microscopic description in Figure 9b. This results in an enlarged driving-force concentration gradient are denoted as $∆C^{p}=C_{2(l)}^{p}-C_{b(l)}$. The turbulence intensity is enhanced to overcome the concentration polarization effect by disrupting the mass-transfer boundary layer of flow characteristics near the membrane surface with the implementation of 3D-printed turbulence promoters.

The concentration polarization coefficient $\gamma_{m}$ was derived as a measure of the relative impact on the mass-transfer rate. Both membrane surface concentrations ($C_{1(g)}$ and ${H_{c}K}_{ex}^{\prime }C_{2(l)}$) convective mass-transfer coefficients ($k_{a}$ and $k_{b}$) were obtained by equating Equations (3) and (5) (say $J_{m}=J_{g}$) and Equations (4) and (5) (say $J_{m}=J_{l}$) according to the microscopic description in Figure 9b, respectively, as follows:

(10) $$C_{a(g)}=C_{1(g)}+\frac{k_{m}}{k_{a}}\left(C_{1\left(g\right)}-{H_{c}K}_{ex}^{\prime }C_{2(l)}\right)$$

(11) $$H_{c}C_{b(l)}={H_{c}K}_{ex}^{\prime }C_{2(l)}-\frac{k_{m}}{k_{b}}\left(C_{1\left(g\right)}-{H_{c}K}_{ex}^{\prime }C_{2(l)}\right)$$

Then, a is a simplified form of $\gamma_{m}$ expressed in terms of the mass-transfer coefficient as

(12) $$\gamma_{m}=\frac{C_{1\left(g\right)}-{H_{c}K}_{ex}^{\prime }C_{2(l)}}{C_{a\left(g\right)}-H_{c}C_{b(l)}}=\frac{1}{\left(1+\frac{k_{m}}{k_{a}}+\frac{k_{m}}{k_{b}}\right)}=\frac{k_{a}k_{b}}{k_{a}k_{b}+k_{m}k_{b}+k_{m}k_{a}}$$

The mass-transfer enhancement factor, $\alpha^{p}$ [52], is defined as the ratio of the mass-transfer rate improvement in a module with embedded turbulence promoters compared to that in a module with an empty channel, under various array configurations. To evaluate the enhancement of the mass-transfer rate by implementing a promoter-filled channel in membrane contactors, comparisons were made between modules with turbulence promoters and those without. Augmented mass-transfer coefficients were related to the mass-transfer enhancement factor and expressed in terms of the correlated Sherwood number. The correlated Sherwood number was integrated into the mass-transfer enhancement factor, $\alpha^{p}$, to improve the mass-transfer coefficient and reduce the concentration polarization effect, thereby increasing the driving force across the membrane. A simple relationship [53] between ${Sh}^{p}$ (the Sherwood number for the turbulence-promoter channel) and ${Sh}_{lam}$ (the Sherwood number for laminar flow in the empty channel) is given by

(13) $${Sh}^{p}=\frac{k_{b}D_{h,promoter}}{D_{b}}=\alpha^{p}{Sh}_{lam}$$

in which, the corelated Sherwood number ${Sh}^{p}$ is defined as the module of inserting various promoter-filled configurations and incorporating dimensionless groups into Buckingham’s π theorem, while the Sherwood number ${Sh}_{lam}$ is the membrane contactor using the no-promoter-filled channel under laminar flow operations, with the regressed correlation equation as

(14) $$\alpha^{p}=\frac{{Sh}^{p}}{{Sh}_{lam}}=f\left(\frac{W_{P}}{L},W_{\mathrm{ratio}}{,Re}_{l},\;{Re}_{g},\;\;\;\;{Sc}_{g}\right)$$

where $W_{P}$ is the average promoter width of inserting turbulence promoters while the $W_{ratio}$ is the ratio of average diameter for various configurations based on the diameter of the big circle-type turbulence promoter.

### 3.2. Mathematical Formulations

**Figure 10:**
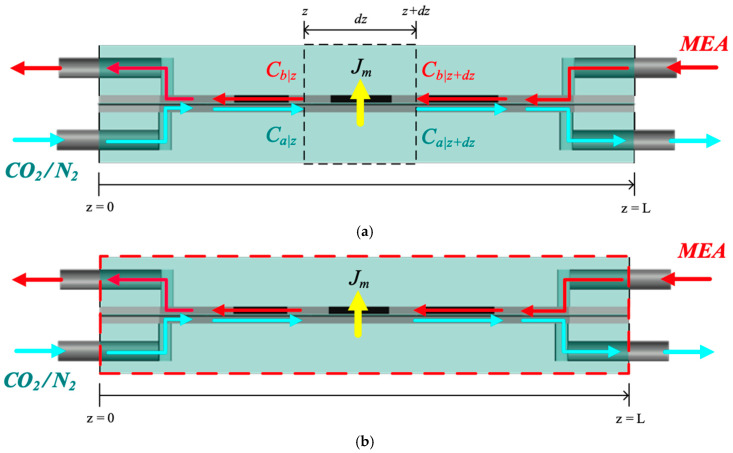
Plug-flow and macroscopic descriptions of mass-transfer mechanisms within the control volume. (**a**) Plug-flow description; (**b**) Macroscopic description.

The one-dimensional mathematical modeling equations were obtained by constructing the mass flux diagram in a finite control element according to the mass conservation law under steady-state operations, with the coordinate along the *z*-axis representing the flow direction. The schematic diagram of the plug-flow description considers only the largest concentration gradient in the mass-transfer balance equation, neglecting all diffusion terms, as shown in Figure 10a. This approach is associated with the most simplified model, represented macroscopically in Figure 10b for both CO_2_/N_2_ and MEA feed streams.

(15) $$\frac{d\left[q_{a}C_{a(g)}\right]}{\mathrm{dz}}=-W\left[k_{m}\gamma_{m}\left(C_{a(g)}-{H_{c}K}_{ex}^{\prime }C_{b(l)}\right)\right]$$

(16) $$\frac{d\left[q_{b}C_{b(l)}\right]}{\mathrm{dz}}=-W\left[k_{m}\gamma_{m}\left(C_{a(g)}-{H_{c}K}_{ex}^{\prime }C_{b(l)}\right)\right]+Wdk_{CO_{2}}C_{b(l)}$$

**Figure 11:**
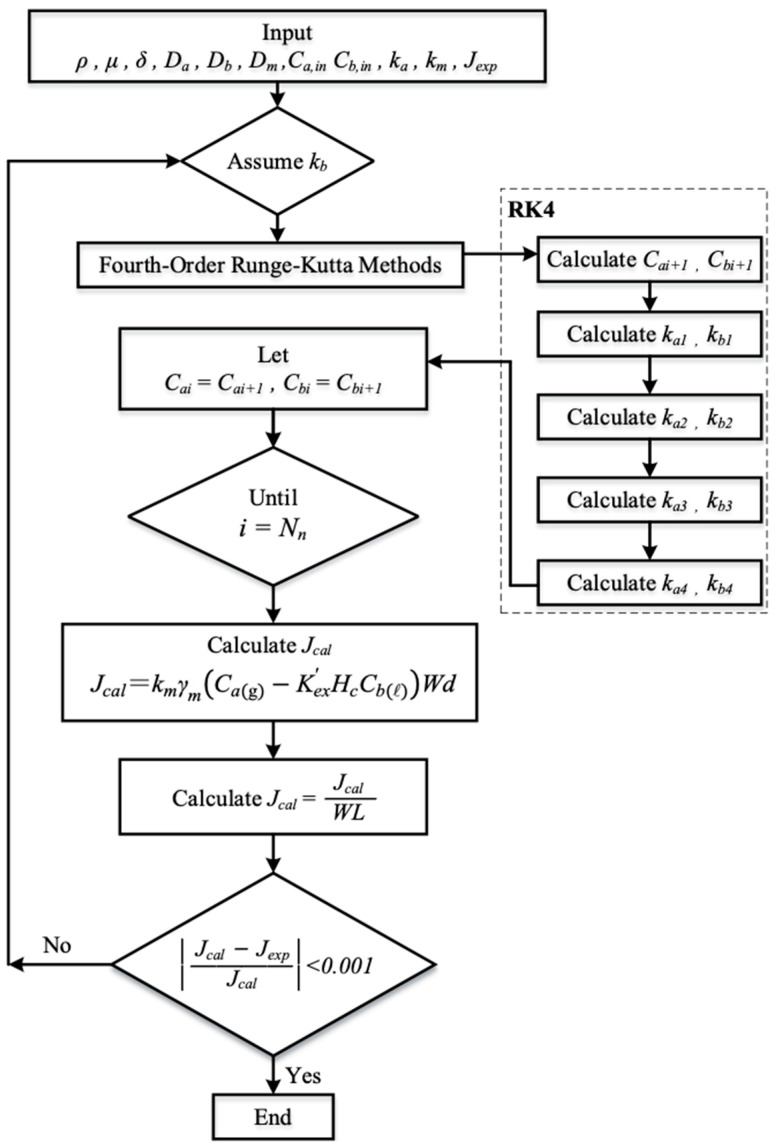
Flow chart for determining CO_2_ concentrations in both gas and liquid phases.

The simultaneous ordinary differential equations, Equations (15) and (16), were solved using estimated convective mass-transfer coefficients. The equations were iteratively calculated along the membrane absorption module in Figure 11 using the fourth-order Runge–Kutta method. This numerical approach was employed to obtain the CO_2_ concentration distributions in the CO_2_/MEA bulk streams, thereby determining the CO_2_ absorption flux and its improvement. The fourth order Runge–Kutta method was chosen to minimize the tradeoff between accuracy and computational efficiency, resulting in the marching solutions of the CO_2_ concentrations in both CO_2_/N_2_ and MEA feed streams and the subsequent determination of CO_2_ absorption flux and its enhancement.

### 3.3. Power Consumption Increment

The present study proposes an investigation into designing promoter-filled channels that act as turbulence promoters to enhance the performance of membrane contactor applications. This design, however, results in an unavoidable increase in energy consumption due to the increased frictional loss. The hydraulic consumption increment is anticipated from both CO_2_/N_2_ and MEA feed streams in the innovative promoter-filled channel. This increment can be determined using the Fanning friction factor ($f_{F}$) [54], considering only the friction losses to the walls of both feed streams in a flat-plate promoter-filled membrane contactor of known length, as follows:

(17) $$H_{i}=q_{a}\;\rho_{{CO}_{2}}\;lw_{f,{CO}_{2}}+q_{b}\;\rho_{MEA}\;\;lw_{f,MEA},\;i=promoter,\;empty$$

(18) $${lw}_{f,\mathrm{MEA}}=\frac{2f_{F,MEA}{\bar{v}}_{MEA}^{2}L}{D_{h,MEA}}$$

(19) $${lw}_{f,{CO}_{2}}=\frac{2f_{F,CO_{2}}{\bar{v}}_{CO_{2}}^{2}L}{D_{h,CO_{2}}}$$

**Figure 12:**
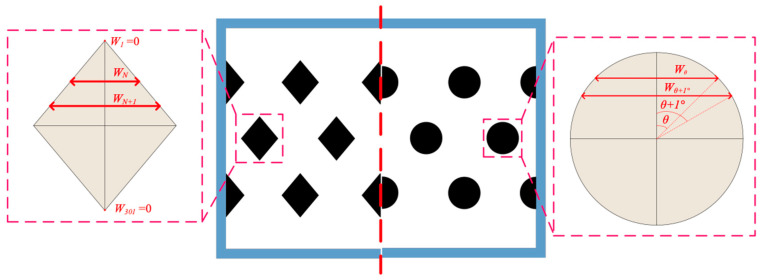
Average widths of geometric diamond-type and circle-type turbulence promoters.

The hydraulic equivalent diameter $D_{h,MEA}$ and $D_{h,CO_{2}}$ of modules with embedded 3D-printed turbulence promoters and empty channels of both MEA and CO_2_/N_2_ feed streams, respectively, was calculated by the wetted area A and wetted perimeter P, say 4A/P, with the use of the average turbulence promoter width of 3D-printed diamond-type and circle-type promoters, as shown in Figure 12, which were evaluated by averaging various segments of the diamond-type and circle-type shapes.

The Fanning friction factor can be estimated using a correlation based on the aspect ratio of the channel (α=d/W) [55]:

(20) $$f_{F,h}=\frac{C}{{Re}_{h}},f_{F,c}=\frac{C}{{Re}_{c}}$$

(21) $$C=24\left(1-1.3553\alpha +1.9467\alpha^{2}-1.7012\alpha^{3}+0.9564\alpha^{4}-0.2537\alpha^{5}\right)$$

The percentage and relative extents $I_{P}$ of the energy consumption increase for the module when inserting the promoter-filled channel was illustrated, in comparison to the module of using the empty channel, as

(22) $$I_{P}=\frac{H_{promoter}-H_{empty}}{H_{empty}}\times 100\%$$

where the subscripts of the *promoter* and *empty* represent the modules using the promoter-filled channel and empty channel, respectively. Further focused research based on economic considerations is needed to evaluate the influence of promoter-filled channels.

## 4. Results and Discussions

### 4.1. Absorption Flux Improvement by Inserting 3D-Printed Turbulence Promoters

Both bulk concentration distributions of CO_2_/N_2_ and MEA feed streams, as well as membrane surface concentrations in membrane absorption modules, were solved numerically using the one-dimensional theoretical model, as presented in Equations (15) and (16) along the axial coordinate with various array configurations. The range and limits of the accuracy deviation between the theoretical predictions and experimental results for all measurements of descending diamond and circle promoters as illustrations was calculated using the following definition of accuracy deviation [56]:

(23) $$E\;(\%)=\frac{1}{N_{exp}}\sum_{j=1}^{N_{exp}}\frac{\left|J_{theo,j}-J_{\exp,j}\right|}{J_{\exp,j}}\;$$

Moffat [56] determined the experimental uncertainty for each individual measurement from the experimental runs as follows:

(24) $$S_{J_{exp}}={\left\{\sum_{i=1}^{N_{exp}}\frac{{\left(J_{exp,i}-\bar{J_{exp,i}}\right)}^{2}}{N_{exp}-1}\right\}}^{1/2}\;\;\;$$

The mean value of the resulting uncertainty of the experimental measurements was defined by

(25) $$S_{\bar{J_{exp}}}=\frac{S_{J_{exp}}}{\sqrt{N_{exp}}}\;\;\;$$

where $N_{exp}$, $J_{theo,j}$, and $J_{\exp,j}$ are the number of experimental runs, theoretical predictions and experimental results of absorption fluxes, respectively. The agreement of experimental results deviated from theoretical predictions and uncertainty of the experimental measurements are well minimized within $8.0\times 10^{-4}\leq E\leq 3.01\times 10^{-2}$ and $5.34\times 10^{-3}\leq S_{\bar{J_{exp}}}\leq 8.23\times 10^{-3}$, respectively. It is seen from Table S1 in the Supporting Information section that good agreement was expected between the theoretical predictions and experimental results.

**Figure 13:**
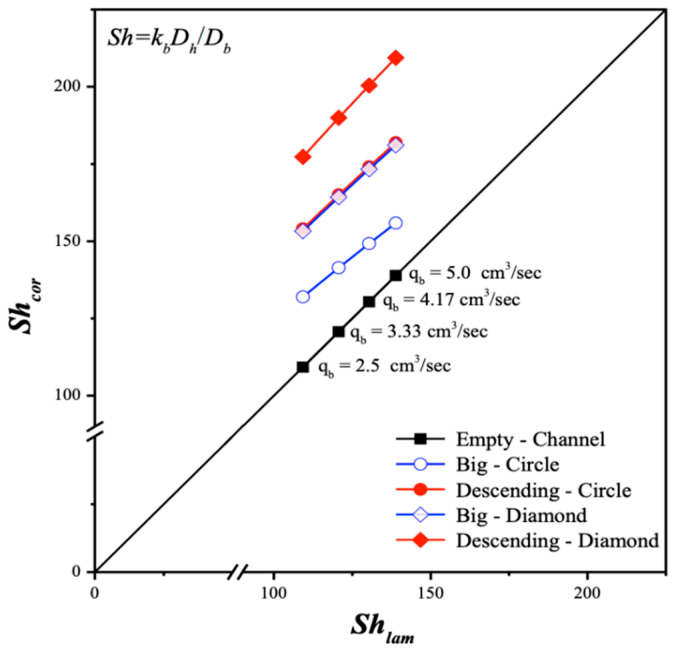
Comparison of correlated and experimental Sherwood numbers for the no-promoter-filled and promoter-filled channels under various array configurations.

Two geometric shapes of 3D-printed turbulence promoters—diamond-type and circle-type—were used in gas/liquid membrane contactors, with six different promoter structures at various average equivalent widths. All promoters occupied approximately 13% of the hydrophobic membrane surface area, allowing for a comparison of CO_2_ absorption fluxes in this study. The incorporation of turbulence promoters aimed to enhance turbulence intensity, effectively reducing the concentration polarization effect on the boundary layer. This was achieved by increasing velocities and vortices, which enhanced shear stress on the membrane surface, ultimately improving CO_2_ absorption efficiency and device performance. Notably, the insertion of diamond-type turbulence promoters, with their non-smooth curvature, led to significantly higher CO_2_ absorption flux compared to the circle-type promoters. This suggests that operating a channel filled with diamond-type promoters generated greater turbulence intensity, which reduced concentration polarization resistance and further enhanced CO_2_ absorption flux. The mass-transfer coefficients, expressed in terms of the correlated Sherwood number, were determined using a theoretical model and compared with a module without promoter-filled channels. The results align linearly with the experimental data, as shown in Figure 13.

**Figure 14:**
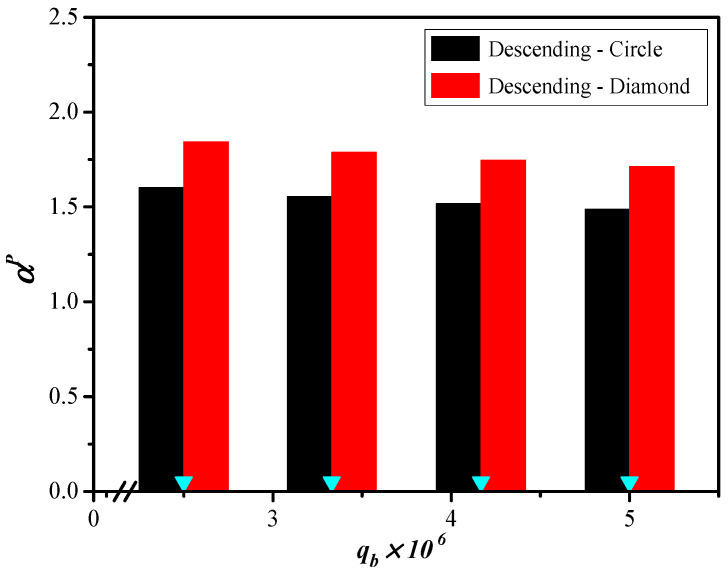
Comparisons of theoretical Sherwood numbers for the channels with inserted 3D-printed turbulence promoters of both diamond and circle types.

The correlation expression of Sherwood numbers is applicable for channels with embedded turbulence promoters as well. Another aspect to consider is the improved device performance of various promoter-filled channel configurations, which disrupt the mass-transfer boundary layer and increase turbulence intensity. The mass-transfer enhancement factor $\alpha^{P}$ was expressed in Equation (14) and correlated via regression analysis by setting up the normal equations of the least squares parameters for modules with promoter-filled channels. This results in enhanced convective mass-transfer efficiency, reflected in a higher Sherwood number compared to the empty channel, as shown in Equation (14) with the use of Equations (26) and (27), and Figure 14.

(26) $${Sh}_{lam}=3.463{\left(\frac{W_{P}}{L}\right)}^{1.751}{Re}_{l}^{0.346}{Re}_{g}^{-3.803}{Sc}_{g}^{1.298}$$

(27) $${Sh}^{P}=(1.612+W_{ratio}){\left(\frac{W_{P}}{L}\right)}^{-1.012}{{Re}_{L}}^{0.240}{{Re}_{G}}^{-2.273}{{Sc}_{G}}^{0.688}$$

**Figure 15:**
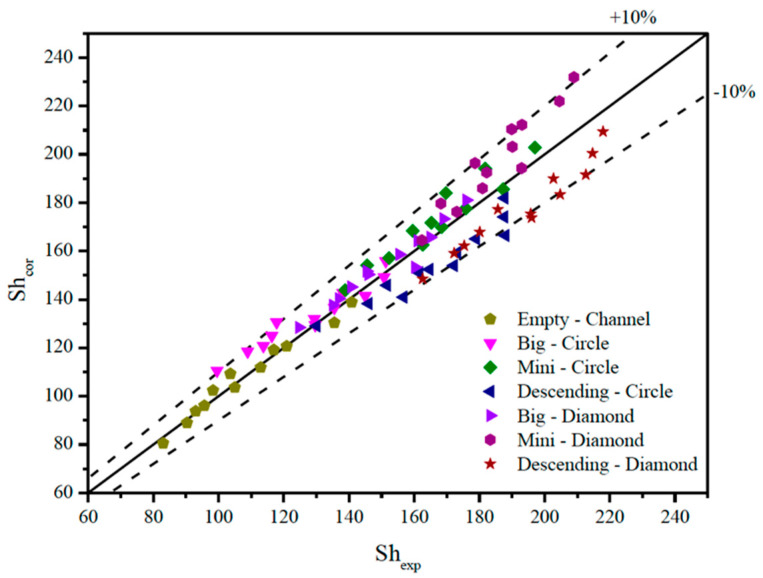
Comparisons between the correlated and experimental Sherwood numbers.

The deviations between the correlated and experimental Sherwood numbers are within 10%, as shown in Figure 15.

**Figure 16:**
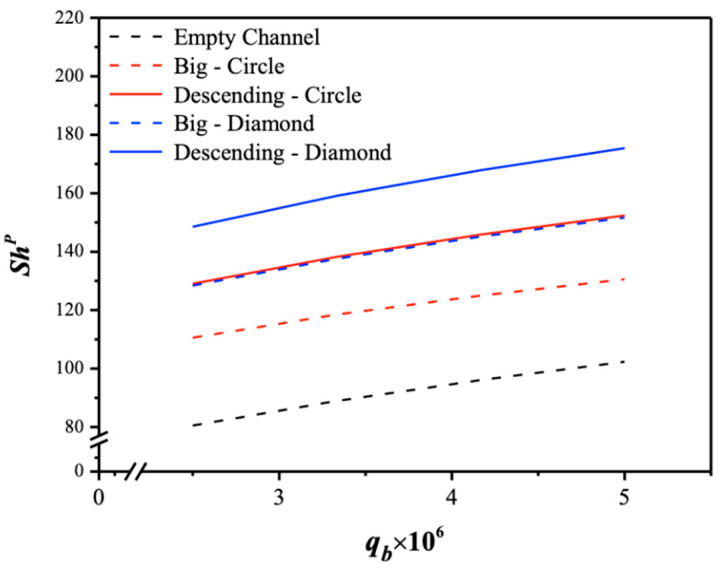
Effects of various array configurations and geometric shapes of 3D-printed turbulence promoters on Sherwood numbers.

The 3D-printed turbulence promoters play a crucial role in disrupting the concentration boundary layer and reducing mass-transfer resistance, thereby increasing the CO_2_ absorption flux. The correlated Sherwood numbers in Equation (27) indicate that the mass-transfer coefficient of the module with descending promoter widths achieves a higher mass-transfer rate than that of the uniform promoter widths and the empty channel. The results also show that the module with diamond-type turbulence promoters enhances vortices and eddies more effectively than the module with circle-type turbulence promoters, due to the non-smooth curvature shape of the obstacles, as depicted in Figure 16.

### 4.2. CO_2_ Absorption Flux Improvement by Embedding Various Turbulence Promoters

**Figure 17:**
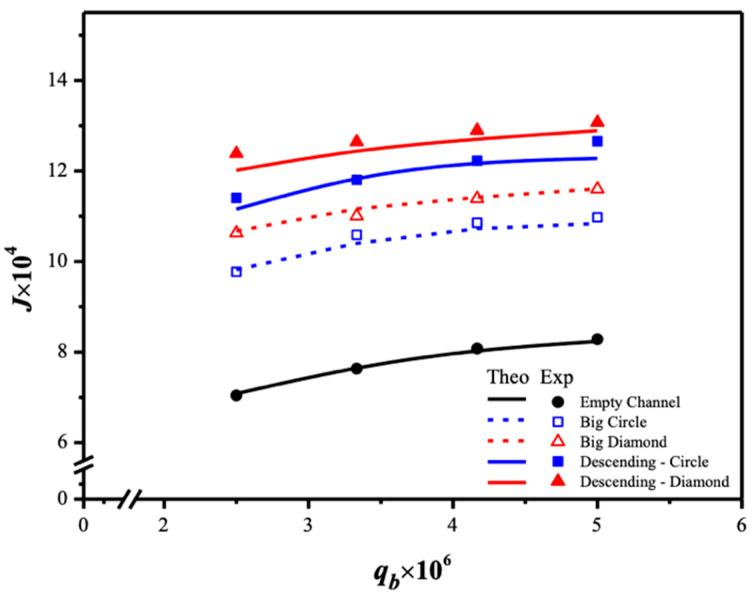
Comparisons of theoretical CO_2_ absorption flux under both descending diamond-type and circle-type turbulence promoters.

The larger driving-force concentration gradient across the membrane surfaces, denoted as $C_{1(g)}$ and ${H_{c}K}_{ex}^{\prime }C_{2(l)}$, as shown in Figure 9b, results in a greater absorption flux passing through the membrane. Consequently, a larger value of the concentration polarization coefficient $\gamma_{m}$ was achieved when the module with 3D-printed turbulence promoters was inserted into the MEA absorbent feed stream, reducing the mass-transfer boundary layer thickness. Introducing various array configurations of promoter-filled channels increased turbulence intensity near the membrane surface, nearly doubling the CO_2_ absorption flux. This study provides a graphical representation comparing theoretical predictions of absorption fluxes obtained in the present study for descending and large-type promoter-filled channels. The preference for the current design of inserting 3D-printed turbulence promoters is illustrated in Figure 17 with a 35% inlet CO_2_ feed concentration as an example. The current study continues to demonstrate better performance by exploring promoter-filled channels in descending diamond and circle shapes, achieving higher absorption flux than uniform promoter-filled channels, as depicted in Figure 17.

**Figure 18:**
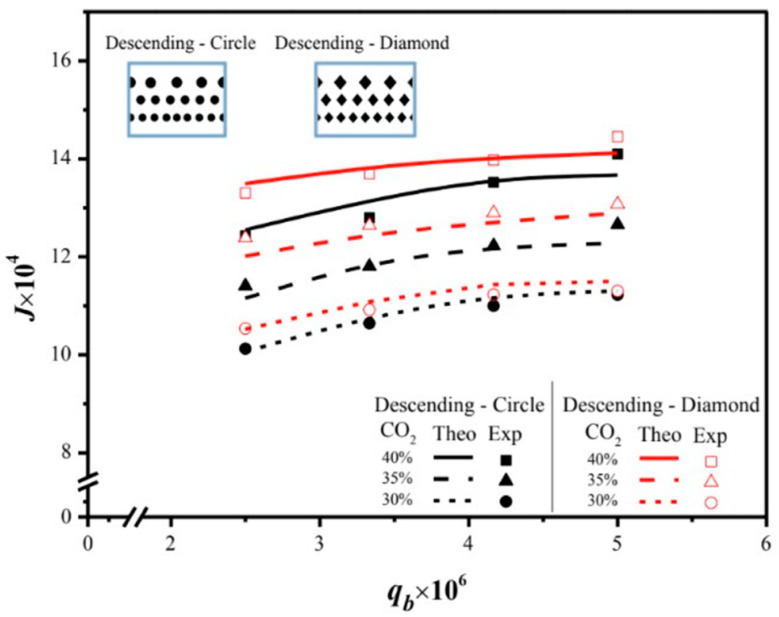
Effects of MEA flow rate and inlet CO_2_ feed concentration on CO_2_ absorption fluxes under both descending diamond- and circle-promoter diameters.

This design emphasizes technical feasibility and demonstrates significantly enhanced absorption flux. The predictive capability for absorption flux can be extended to various geometric promoters and array configurations by following the same regression procedure applied to Sherwood numbers for both modules with an empty channel and those with inserted 3D-printed turbulence promoters. The results indicate that higher inlet CO_2_ feed concentrations lead to larger ${Sh}^{p}$ numbers, resulting in a higher mass-transfer rate. Both experimental findings and theoretical predictions of absorption fluxes are presented graphically, utilizing inlet CO_2_ feed concentrations and MEA absorbent feed flow rates as parameters. This representation is outlined in Figure 18 for operating both uniform and descending promoter-filled channels, considering diamond-type and circle-type turbulence promoters, respectively. The agreement between theoretical predictions and experimental data are fairly consistent, providing a solid basis for evidence-based justification, as illustrated in Figure 18. A slight concentration polarization effect is observed in the first half of the module compared to a notable concentration polarization effect in the latter half of the module, leading to higher absorption flux in modules using descending promoter-filled channels compared to those employing uniform big-promoter-filled channels. Moreover, Figure 18 illustrates the order of absorption fluxes among membrane absorption modules utilizing promoter-filled channels: descending diamond-type promoters > descending circle-type promoters. CO_2_ absorption fluxes of all array configurations increase with higher inlet CO_2_ feed concentrations but decrease with higher MEA feed flow rates.

**Figure 19:**
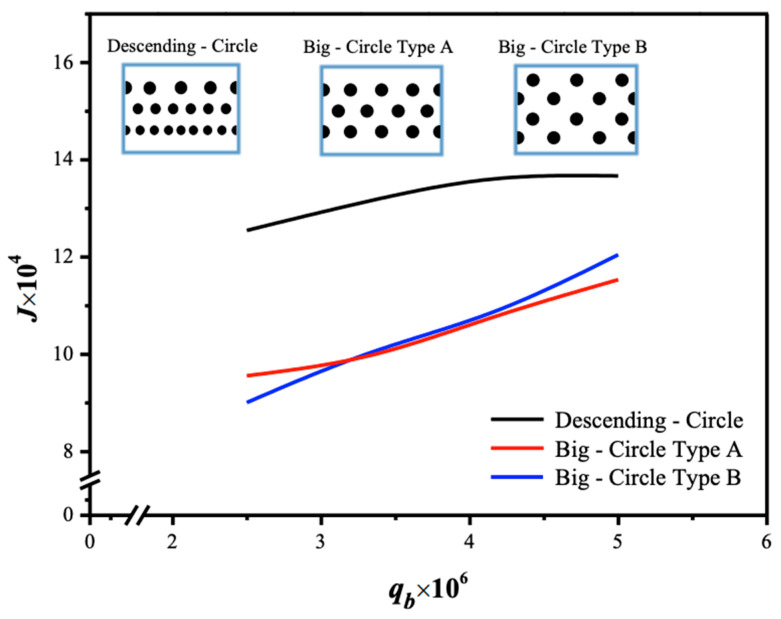
Comparisons of theoretical CO_2_ absorption flux of the modules when implementing descending promoter-filled channels and uniform promoter-filled channels.

The present work extends the previous study by embedding 3D-printed turbulence promoters into descending promoter-filled channels, as opposed to using uniform promoter-filled channels [45]. Comparisons between descending promoter-filled-channel and uniform promoter-filled-channel modules [45] demonstrate why the descending promoter-filled-channel design is preferred, as illustrated in Figure 19 for both configurations. This highlights the value and originality of the present study, particularly regarding its technical feasibility.

It is noteworthy that a higher CO_2_ absorption flux was achieved at a higher inlet CO_2_ feed concentration, with the order of absorbent flux magnitude being 40% > 35% > 30%. This was attributed to the greater absorption flux emerging due to more intensive vortices and eddies, resulting in a higher concentration gradient between both sides of the membrane. This effect is particularly pronounced with mini-type turbulence promoters under the same total coverage area, mitigating the mass-transfer boundary layer. The relative absorption flux improvements $I_{N}$ were illustrated by calculating the percentage increment in comparisons between the absorption flux of the module using an empty channel and those inserting 3D printing turbulence promoters as shown below.

(28) $$I_{N}(\%)=\frac{J_{promoter}-J_{empty}}{J_{empty}}\times 100,\mathrm{module}\;\mathrm{with}\;\mathrm{promoter}\text{-}\mathrm{filled}\;\mathrm{channels}$$

where the subscripts promoter and empty represent the channels with/without inserting turbulence promoter, respectively.

The theoretical predictions of the CO_2_ absorption flux improvements $I_{N}$ in the module with inserted descending and uniform promoter widths are summarized in Tables S2–S4 of the Supporting Information section, with inlet CO_2_ feed concentration and MEA feed flow rate as parameters. The analysis reveals that the increased concentration gradients facilitate greater mass diffusion through the membrane, consequently leading to a higher amount of absorption flux in the MEA absorbent feed stream with inserted 3D-printed turbulence promoters compared to those in the empty channel. The insertion of 3D-printed turbulence promoters into the flow channel substantially augments absorption flux by reducing the concentration polarization effect. The device performance of mini-promoter-filled channels is superior to that of big-promoter-filled channels, as confirmed by Tables S2–S4 of the Supporting Information section. The module with descending diamond-promoter-filled channels exhibits a relative increment in absorption flux of up to 79.12% under a 40% inlet CO_2_ feed concentration and a 2.5 cm^3^/s MEA feed flow rate compared to the no-promoter-filled module, as confirmed by Table S4 of the Supporting Information section. The undesirable influence on the CO_2_ absorption flux, transferring from the gas side to the MEA feed stream, was reduced by embedding various shapes and array configurations of 3D-printed turbulence promoters. This effect is expressed in terms of the concentration polarization coefficient, $\gamma_{m}$. The insertion of turbulence promoters enhanced the absorption flux by disrupting the concentration polarization layers, leading to a higher $\gamma_{m}$ value and improved absorption flux. Theoretical predictions of the concentration polarization coefficients show that a larger $\gamma_{m}$ value was achieved in the descending promoter-filled-channel modules compared to the uniform promoter-filled-channel modules.

### 4.3. Energy Consumption Increment

**Figure 20:**
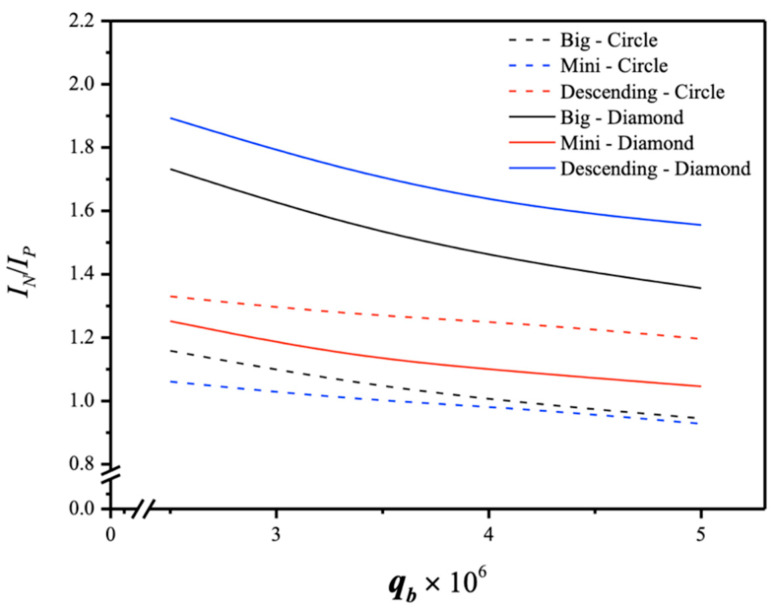
Comparisons of $I_{N}/I_{P}$ with inserted 3D-printed turbulence promoters under various array configurations.

From an economic viewpoint, assessing the suitable selection of operation conditions for both absorption flux enhancement and energy consumption increment, represented by $I_{N}/I_{P}$, was crucial for the modules with promoter-filled channels. The study examined technical feasibilities considering the trade-offs of additional friction losses. A higher ratio of $I_{N}/I_{P}$ was achieved by appropriately arranging the array configurations of turbulence promoters, particularly with descending promoter-filled channels, which could accomplish more effective absorption flux at the expense of energy consumption, as shown in Figure 20, with MEA flow rates and promoter configurations as parameters. The results demonstrated that increasing the CO_2_ absorption flux could not compensate for the power consumption increment by merely increasing the MEA flow rate. Superior device performance was observed with diamond-promoter-filled channels under descending array configurations; specifically, the descending diamond-promoter module outperformed the descending circle-promoter module, as well as uniform big-promoter and mini-promoter modules. In summary, the percentage increment of absorption flux improvement was higher than the percentage increment of energy consumption. Essentially, embedding 3D-printed turbulence promoters with descending promoter shapes in the MEA absorbent feed channel achieved desirable absorbent flux enhancement while counterbalancing the undesirable friction loss increment.

Notably, the mini-promoter channel induces higher turbulence intensity, resulting in reduced mass-transfer resistance. The comparison reveals that a higher absorption flux $I_{N}$ is achieved when operating the module with uniform mini-promoter-filled channels than with descending and big promoter-filled channels, in the order of mini promoters > descending promoters > big promoters. By contrast, the ratio of $I_{N}/I_{P}$ for various promoter-filled configurations changes, favoring the modules with descending diamond-promoter-filled and circle-promoter-filled channels, in the order of descending promoters > mini promoters > big promoters, as shown in Figure 20. In other words, the descending promoter-filled channel can increase CO_2_ absorption flux more effectively than the uniform promoter-filled channel when considering the energy consumption increment from an economic viewpoint.

Furthermore, the reverse order happened for $I_{N}$ and $I_{N}/I_{P}$ in operating uniform big-type, and the descending promoter-filled channels were examined by the further CO_2_ absorption flux enhancement $E_{P}$ of CO_2_ capture by operating descending promoter-filled channels, which calculated the enhancement based on the device of the same working dimensions performed under the uniform promoter-filled channels as follows:

(29) $$\begin{array}{cc} E_{P}\left(\%\right) & =\frac{J_{promoter}^{des}-J_{promoter}^{uni}}{J_{promoter}^{uni}}\times 100\\ & =\left[\frac{\left(J_{promoter}^{des}-J_{empty})-(J_{promoter}^{uni}-J_{empty}\right)}{J_{empty}}\right]\left(\frac{J_{empty}}{J_{promoter}^{uni}}\right)\times 100=\left(I_{N}^{des}-I_{N}^{uni}\right)/\left(1+I_{N}^{uni}\right)\times 100 \end{array}$$

where $J_{promoter}^{uni}$ and $J_{promoter}^{des}$ are the absorption fluxes in the module with embedding uniform and descending promoter-filled channels for both circle-type and diamond-type, respectively. Meanwhile $I_{N}^{uni}$ and $I_{N}^{des}$ are the absorption flux improvements in the module with embedding uniform and descending promoter-filled channels for both circle-type and diamond-type, respectively. A percentage increment of absorption flux improvement and further absorption flux enhancement was evaluated for the module with embedded circle-type and diamond-type promoter-filled channels, respectively, as seen in Table S5. Further absorption flux enhancement is accomplished by embedding descending promoter-filled channels into the MEA feed stream, thereby increasing the convective mass-transfer coefficient. This enhancement increases with inlet CO_2_ feed concentration but decreases with MEA feed flow rate. A maximum 20.02% further absorption flux enhancement is achieved with diamond-type promoter-filled channels, more than the same device with circle-type promoter-filled channels, as seen in Table S5 of the Supporting Information section. As anticipated, descending promoter-filled channels yield a more significant enhancement in absorption flux compared to uniform promoter-filled channels, resulting in the reverse order for $I_{N}/I_{P}$ when operating uniform mini-type and descending promoter-filled channels.

## 5. Conclusions

The theoretical predictions of absorption flux were calculated and validated by experimental results under various MEA feed flow rates, inlet CO_2_ feed concentrations, and array configurations for both diamond and circle turbulence promoter-filled channels. Comparisons of absorption flux improvement, by inserting 3D-printed turbulence promoters into flow channels, leads to the following conclusions:

1. Inserting 3D-printed diamond turbulence promoters into the MEA feed stream results in relative increases in absorption flux, with a maximum improvement of 79.12% under a descending array configuration compared to the module using an empty channel.
2. The results demonstrate that promoter-filled channels with descending promoter widths in membrane absorption modules achieve a more pronounced absorption flux improvement $I_{N}$ due to a larger driving-force concentration gradient.
3. The study shows a higher absorption flux improvement $I_{N}$ in modules with uniform mini-promoter-filled channels compared to those with descending promoter-filled channels and uniform big-promoter-filled channels. However, the ratio of $I_{N}/I_{P}$ for descending promoter-filled channels follows a reverse order.
4. Embedding promoter-filled channels with descending promoter widths into the MEA flow channel results in absorption flux improvement of up to 79.12% under a 40% inlet CO_2_ feed concentration and a 2.5 cm^3^/s MEA feed flow rate.

The correlated equation of the Sherwood number, derived from the theoretical mathematical model, provides valuable insights for designing more efficient membrane absorption modules. While this study specifically focuses on evaluating absorption flux improvement and energy consumption increment by inserting 3D-printed turbulence promoters into the MEA absorbent feed channel, further investigation is needed to explore alternative geometric shapes and array configurations of 3D-printed promoter-filled channels for optimal operation, considering the economic viewpoint.

## Data Availability

Data is contained within the article.

## Supplementary Materials

The following supporting information can be downloaded at: https://www.mdpi.com/article/10.3390/membranes15030088/s1, Table S1. The accuracy deviation between theoretical predictions $J_{theo}$ and experimental results $J_{exp}$ of absorption fluxes. Table S2. Effects of promoter-filled operations on absorption flux improvements $I_{N}.$ Table S3. Effects of promoter-filled operations on absorption flux improvements $I_{N}.$ Table S4. Effects of promoter-filled operations on absorption flux improvements $I_{N}.$ Table S5. Theoretical predictions of the absorption flux improvement $I_{N}$ and further absorption flux enhancement $E_{P}$ in the module with descending promoter-filled channels.

## Nomenclature

C	Concentration (mol m^−3^)
$C_{mean}$	Mean value of C (mol m^−3^)
$c_{K}$	Membrane coefficient based on the Knudsen diffusion model (mol m^−2^ pa^−1^ s^−1^)
$c_{M}$	Membrane coefficient based on the molecular diffusion model (kg m^−2^ Pa^−1^ s^−1^)
$c_{m}$	Membrane permeation coefficient (mol m^−2^ pa^−1^ s^−1^)
$D_{a}$	Diffusion coefficient of CO_2_ in gas feed stream (m^2^ s^−1^)
$D_{b}$	Diffusion coefficient of CO_2_ in MEA absorbent feed stream (m^2^ s^−1^)
d	Channel thickness (m)
$d_{h,i}$	Equivalent hydraulic diameter of channel (m), i=spiral, empty
E	Deviation of experimental results from the theoretical predictions
$E_{P}$	Further absorption flux enhancement
$f_{F}$	Fanning friction factor
$H_{c}$	Dimensionless Henry’s constant
$H_{i}$	Hydraulic dissipate energy (J kg^−1^), i=spiral, empty
$I_{N}$	Absorption flux improvement
$I_{P}$	Power consumption relative index
$k_{a}$	Mass transfer coefficient in the CO_2_/N_2_ gas feed stream (m s^−1^)
$k_{b}$	Mass transfer coefficient in the MEA absorbent feed stream (m s^−1^)
$K_{ex}$	Equilibrium constant
$K_{ex}^{\prime }$	Reduced equilibrium constant
$K_{m}$	Overall mass transfer coefficient of membrane (m s^−1^)
${lw}_{f,i}$	Friction loss (J kg^−1^), $i=CO_{2}/N_{2},\;MEA$
L	Channel length (m)
$M_{w}$	Average molecular weight of CO_2_ and N_2_ gas mixture (${kg\;mol}^{-1}$)
$N_{exp}$	Number of experimental measurements
$P_{1}$	Saturation vapor pressure in the gas feed flow side (Pa)
$P_{2}$	Saturation vapor pressure in the liquid absorbent flow side (Pa)
$q_{a}$	Volumetric flow rate of the gas feed stream ($m^{3}\;s^{-1}$)
$q_{b}$	Volumetric flow rate of the MEA absorbent stream ($m^{3}\;s^{-1}$)
R	Gas constant (8.314 $J{mol}^{-1}\;K^{-1}$)
Re	Reynolds number
$S_{N_{exp}^{\prime \prime }}$	Precision index of an experimental measurements of absorption flux (mol m^−2^ s^−1^)
$S_{\bar{N_{exp}^{\prime \prime }}}$	Mean value of $S_{N_{exp}^{\prime \prime }}$(mol m^−2^ s^−1^)
Sc	Dimensionless Schmidt number
${Sh}^{P}$	Enhanced dimensionless Sherwood number
${Sh}_{lam}$	Dimensionless Sherwood number for laminar flow
$W_{p}$	Spiral-ring pitch width (m)
$W_{ratio}$	Ratio of the various promoter widths inserted
J	Absorption flux ($mol\;m^{-2}\;s^{-1}$)
${\left|Y_{m}\right|}_{ln}$	Natural log mean CO_2_ mole fraction in the membrane
z	Axial coordinate along the flow direction (m)
Greek letters	
$\alpha^{P}$	Mass-transfer enhancement factor
β	Aspect ratio of the channel
$\delta_{m}$	Thickness of membrane (µm)
ε	Membrane porosity
$\bar{v}$	Average velocity ($m\;s^{-1}$)
$\rho_{i}$	Density ($kg\;m^{-3}$), $i=CO_{2}/N_{2},\;MEA$
$\gamma_{m}$	Concentration polarization coefficients
Subscripts	
1	Membrane surface in CO_2_/N_2_ gas feed side
2(l)	Liquid phase on membrane surface on MEA feed stream
2(g)	Gas phase on membrane surface on MEA side
*a*	The CO_2_ /N_2_ gas feed stream
*b*	The MEA absorbent feed stream
*cor*	Correlated results
*promoter*	Inserting spiral rings as promoters
*empty*	Empty channel
*exp*	Experimental results
g	Gas feed stream
*in*	At the inlet
l	MEA absorbent feed stream
*out*	At the outlet
*theo*	Theoretical predictions
Superscripts	
*uni*	Uniform promoter widths
*des*	Descending promoter widths

## References

[1] Li R., Xu J., Wang L., Li J., Sun X. (2009). Reduction of VOC emissions by a membrane-based gas absorption process. J. Environ. Sci..

[2] Eide-Haugmo I., Lepaumier H., Einbu A., Vernstad K., da Silva E.F., Svendsen H.F. (2011). Chemical stability and biodegradability of new solvents for CO_2_ capture. Energy Procedia.

[3] Herzog H., Eliasson B., Kaarstad O. (2000). Capturing greenhouse gases. Sci. Am..

[4] Dowell N.M., Fennell P.S., Shah N., Maitland G.C. (2017). The role of CO_2_ capture and utilization in mitigating climate change. Nat. Clim. Change.

[5] Mangalapally H.P., Notz R., Hoch S., Asprion N., Sieder G., Garcia H., Hasse H. (2009). Pilot plant experimental studies of post combustion CO_2_ capture by reactive absorption with MEA and new solvents. Energy Procedia.

[6] Aaron D., Tsouris C. (2005). Separation of CO_2_ from flue gas: A review. Sep. Sci. Technol..

[7] Sea B., Park Y.I. (2002). Comparison of porous hollow fibers as a membrane contactor for carbon dioxide absorption. J. Ind. Eng. Chem..

[8] Li J.L., Chen B.H. (2005). Review of CO_2_ absorption using chemical solvents in hollow fiber membrane contactors. Sep. Purif. Technol..

[9] Takahashi N., Furuta Y., Fukunaga H., Takatsuka T., Mano H., Fujioka Y. (2011). Effects of membrane properties on CO_2_ recovery performance in a gas absorption membrane contactor. Energy Procedia.

[10] Klaassen R., Feron P., Jansen A. (2008). Membrane contactor applications. Desalination.

[11] Wang W.P., Lin H.T., Ho C.D. (2006). An analytical study of laminar co-current flow gas absorption through a parallel-plate gas-liquid membrane contactor. J. Membrane Sci..

[12] Ghasem N., Al-Marzouqi M., Duidar A. (2012). Effect of PVDF concentration on the morphology and performance of hollow fiber membrane employed as gas-liquid membrane contactor for CO_2_ absorption. Sep. Purif. Technol..

[13] Zhang C.Y., Hu H.C., Chai X.S., Pan L., Xiao X.M. (2013). A novel method for the determination of adsorption partition coefficients of minor gases in a shale sample by headspace gas chromatography. J. Chromatogr. A.

[14] Xu P., Qiu M.H., Fu K.Y., Chen X.F., Fan Y.Q. (2023). Enhancing performance of ceramic membrane in CO_2_ membrane absorption: Single-to multi-channel. J. Membr. Sci..

[15] Lee H.J., Kim M.K., Park J.H., Magnone E. (2020). Temperature and pressure dependence of the CO_2_ absorption through a ceramic hollow fiber membrane contactor module. Chem. Eng. Process. Process Intensif..

[16] Scholesa C.A., Kentisha S.E., Stevensa G.W., deMontigny D. (2015). Comparison of thin film composite and microporous membrane contactors for CO_2_ absorption into monoethanolamine. Inter. J. Greenh. Gas. Control.

[17] Bottion A., Capannelli G., Comite A., Felice R., Firpo R. (2008). CO_2_ removal from a gas stream by membrane contactor, Sep. Purif. Technol..

[18] Rochelle G.T. (2009). Amine Scrubbing for CO_2_ Capture. Science.

[19] Lepaumier H., Picq D., Carrette P.L. (2009). Degradation Study of New Solvents for CO_2_ Capture in Post-Combustion. Energy Procedia.

[20] Tobiesen F.A., Svendsen H.F. (2006). Study of a modified amine-based regeneration unit. Ind. Eng. Chem. Res..

[21] Ho M.T., Allinson G.W., Wiley D.E. (2011). Comparison of MEA capture cost for low CO_2_ emissions sources in Australia. Int. J. Greenh. Gas Control.

[22] Lee H.J., Park Y.G., Kim M.K., Lee S.H., Park J.H. (2019). Study on CO_2_ absorption performance of lab-scale ceramic hollow fiber membrane contactor by gas/liquid flow direction and module design. Sep. Purif. Technol..

[23] Hamimour N., Sandall O.C. (1984). Absorption of carbon dioxide into aqueous methyldiethanolamine. Chem. Eng. Sci..

[24] Harun N., Nittaya T., Douglas P.L., Croiset E., Ricardez-Sandoval L.A. (2012). Dynamic simulation of MEA absorption process for CO_2_ capture from power plants. Int. J. Greenh. Gas. Control.

[25] Bandini S., Gostoli C., Sarti G.C. (1991). Role of heat and mass transfer in membrane distillation process. Desalination.

[26] Lawson K.W., Lloyd D.R. (1996). Membrane distillation II: Direct contact membrane distillation. J. Membr. Sci..

[27] Ichiyanagi M., Tsutsui I., Kakinuma Y., Sato Y., Hishida K. (2012). Three-dimensional measurement of gas dissolution process in gas–liquid microchannel flow. Int. J. Heat. Mass. Transfer.

[28] Rongwong W., Boributh S., Assabumrungrat S., Laosiripojana N., Jiraratananon R. (2012). Simultaneous absorption of CO_2_ and H_2_S from biogas by capillary membrane contactor. J. Membr. Sci..

[29] Sefidi V.S., Winand I., Luis P. (2023). Enhanced carbon dioxide membrane-based absorption with amino acid solutions. J. Chem. Technol. Biotechnol..

[30] Aronu U.E., Svendsen H.F., Hoff K.A. (2010). Investigation of amine amino acid salts for carbon dioxide absorption. Int. J. Greenh. Gas. Control.

[31] Meng J., Li P., Cao B. (2019). High-flux direct-contact pervaporation membranes for desalination. Appl. Mater. Interfaces.

[32] Martínez-Díez L., Vázquez-González M.I., Florido-Díaz F.J. (1998). Study of membrane distillation using channel spacers. J. Membr. Sci..

[33] Chang H., Hsu J.A., Chang C.L., Ho C.D., Cheng T.W. (2017). Simulation study of transfer characteristics for spacer-filled membrane distillation desalination modules. Appl. Energy.

[34] Nasim Afza K., Hashemifard S.A., Abbasi M. (2018). Modelling of CO_2_ absorption via hollow fiber membrane contactors: Comparison of pore gas diffusivity models. Chem. Eng. Sci..

[35] Ho C.D., Chen L., Chen L., Huang M.C., Lai J.Y., Chen Y.A. (2017). Distillate flux enhancement in the air gap membrane distillation with inserting carbon-fiber spacers. Sep. Sci. Technol..

[36] Yeh H.M. (2004). Enrichment of heavy water in rotated wired concentric-tube thermal diffusion columns. Sep. Purif. Technol..

[37] Yeh H.M. (2004). Recovery of deuterium from water–isotopes mixture in concentric-tube thermal diffusion columns inserted with wire spiral for improved performance. Int. J. Hydrogen Energy.

[38] Yeh H.M., Dong J.F., Hsieh M.J., Yang C.C. (2002). Prediction of permeate flux for ultrafiltration in wire–rod tubular-membrane modules. J. Membr. Sci..

[39] Hosseinzadeh A., Hosseinzadeh M., Vatania A., Mohammadi T. (2017). Mathematical modeling for the simultaneous absorption of CO_2_ and SO_2_ using MEA in hollow fiber membrane contactors. Chem. Eng. Process..

[40] Shakaib M., Hasani S.M.F., Mahmood M. (2009). CFD modeling for flow and mass transfer in spacer-obstructed membrane feed channels. J. Membr. Sci..

[41] Taamneh Y., Bataineh K. (2017). Improving the performance of direct contact membrane distillation utilizing spacer-filled channel. Desalination.

[42] Ho C.D., Chang H., Tu J.W., Lim J.W., Chiou C.P., Chen Y.J. (2022). Theoretical and Experimental Studies of CO_2_ absorption in double-unit flat-plate membrane contactors. Membranes.

[43] Santos J.L.C., Geralds V., Velizarov S., Crespo J.G. (2007). Investigation of flow patterns and mass transfer in membrane module channels filled with flow-aligned spacers using computational fluid dynamics (CFD). J. Membr. Sci..

[44] Yeh H.M. (2004). Effect of gradually varying baffled-ring distance on ultrafiltration in tubular membranes inserted concentrically with a ring rod. Desalination Water Treat..

[45] Abueidda D.W., Dalaq A.S., Abu Al-Rub R.K., Younes H.A. (2015). Finite element predictions of effective multifunctional properties of interpenetrating phase composites with novel triply periodic solid shell architectured reinforcements. Int. J. Mech. Sci..

[46] Lee H.J., Binns M., Park S.J., Magnone E., Park J.H. (2019). An experiment and model of ceramic (alumina) hollow fiber membrane contactors for chemical absorption of CO_2_ in aqueous monoethanolamine (MEA) solutions. Korean J. Chem. Eng..

[47] Ding Z.W., Ma R.Y., Fane A.G. (2003). A new model for mass transfer in direct contact membrane distillation. Desalination.

[48] Bhattacharya S., Hwang S.T. (1997). Concentration polarization, separation factor, and Peclet number in membrane processes. J. Membr. Sci..

[49] Zheng Q., Dong L., Chen J., Gao G., Fei W. (2010). Absorption solubility calculation and process simulation for CO_2_ capture. J. Chem. Ind. Eng..

[50] Iversen S.B., Bhatia V.K., Dam-Jphasen K., Jonson G. (1997). Characterization of microporous membranes for use in membrane contactors. J. Membr. Sci..

[51] Lawson K.W., Lloyd D.R. (1997). Membrane distillation. J. Membr. Sci..

[52] Shakaib M., Hasani S.M.F., Mahmood M. (2003). Effects of net-type spacers on heat and mass transfer in direct contact membrane distillation and comparison with ultrafiltration studies. J. Membr. Sci..

[53] Lin S.H., Tung K.L., Chang H.W., Lee K.R. (2009). Influence of Fluorocarbon Fat-Membrane Hydrophobicity on Carbon Dioxide Recovery. Chemosphere.

[54] Welty J.R., Wick C.E., Wilson R.E. (1984). Fundamentals of Momentum, Heat, and Mass Transfer.

[55] Kakac S., Shah R.K., Aung W. (1987). Handbook of Single-Phase Convective Heat Transfer.

[56] Moffat R.J. (1988). Describing the uncertainties in experimental results. Exp. Therm. Fluid. Sci..

