# Supplementary material for: Analysis of CO2 Absorption in Gas/Liquid Membrane Contactors with Inserted Descending Hydraulic Diameters of 3D-Printed Turbulence Promoters
Source: Membranes (Basel). 2025 Mar 9;15(3):88. doi: 10.3390/membranes15030088 (PMC11944136; doi:10.3390/membranes15030088)
Supplement: Supplementary file 1 [file membranes-15-00088-s001.zip › membranes-3318744-supplementary.pdf]

Supporting information

Table S1. The accuracy deviation between theoretical predictions J_{theo} and experimental results J_{exp} of absorption fluxes.

C_{in} (%)	$q_b \times 10^6$ m³/s	Promoter-filled channel configurations with descending hydraulic diameters: Circle type $J_{exp} \times 10^4$ (mol m^{-2} s^{-1})	Circle type $J_{theo} \times 10^4$ (mol m^{-2} s^{-1})	Circle type E (%)	Diamond type $J_{exp} \times 10^4$ (mol m^{-2} s^{-1})	Diamond type $J_{theo} \times 10^4$ (mol m^{-2} s^{-1})	Diamond type E (%)
30	2.5	10.12	10.06	0.60	10.54	10.53	0.08
	3.33	10.64	10.79	1.42	10.92	11.09	1.53
	4.17	11.00	11.22	2.02	11.23	11.44	1.87
	5.0	11.23	11.31	0.76	11.30	11.50	1.79
35	2.5	11.40	11.16	2.14	12.39	12.02	2.98
	3.33	11.80	11.90	0.82	12.64	12.48	1.33
	4.17	12.23	12.21	0.15	12.90	12.71	1.42
	5.0	12.65	12.28	2.96	13.07	12.89	1.37
40	2.5	12.42	12.55	1.04	13.30	13.50	1.47
	3.33	12.80	13.16	2.87	13.69	13.84	1.06
	4.17	13.52	13.61	0.69	13.97	14.03	0.40
	5.0	14.10	13.67	3.01	14.45	14.12	2.31

Table S2. Effects of promoter-filled operations on absorption flux improvements I_N.

C_{in} (%)	$q_b \times 10^6$ (m³ s^{-1})	Promoter-filled channel configurations with uniform big-type promoters: Empty channel $J_{empty} \times 10^4$ (mol m^{-2} s^{-1})	Circle type $J_{circle} \times 10^4$ (mol m^{-2} s^{-1})	Circle type I_N (%)	Diamond type $J_{diamond} \times 10^4$ (mol m^{-2} s^{-1})	Diamond type I_N (%)
30	2.5	6.70	8.81	31.48	9.61	43.40
	3.33	7.39	9.55	29.20	10.16	37.50
	4.17	7.90	9.96	25.96	10.60	34.11
	5.0	8.08	10.07	24.62	10.67	32.05
35	2.5	7.08	9.82	38.68	10.68	50.77
	3.33	7.68	10.40	35.39	11.17	45.32
	4.17	8.06	10.73	33.18	11.42	41.76
	5.0	8.23	10.84	31.66	11.61	40.99
40	2.5	7.53	10.46	38.80	11.77	56.14
	3.33	8.00	11.05	38.08	12.08	51.00
	4.17	8.33	11.47	37.79	12.51	50.21
	5.0	8.48	11.67	37.59	12.68	49.48

Table S3. Effects of promoter-filled operations on absorption flux improvements I_N.

C_{in} (%)	$q_b \times 10^6$ (m³ s^{-1})	Promoter-filled channel configurations with uniform mini-type promoters: Empty channel $J_{empty} \times 10^4$ (mol m^{-2} s^{-1})	Circle type $J_{circle} \times 10^4$ (mol m^{-2} s^{-1})	Circle type I_N (%)	Diamond type $J_{diamond} \times 10^4$ (mol m^{-2} s^{-1})	Diamond type I_N (%)
30	2.5	6.70	10.25	52.98	10.53	57.18

	3.33	7.39	10.95	48.15	11.09	50.16
	4.17	7.90	11.43	44.63	11.48	45.25
	5.0	8.08	11.56	43.11	11.56	43.06
35	2.5	7.08	11.37	60.54	12.48	76.28
	3.33	7.68	11.99	56.09	12.99	69.10
	4.17	8.06	12.32	52.90	13.26	64.61
	5.0	8.23	12.47	51.43	13.34	61.99
40	2.5	7.53	12.75	69.17	13.60	80.46
	3.33	8.00	13.32	66.42	13.94	74.18
	4.17	8.33	13.72	64.81	14.26	71.31
	5.0	8.48	13.73	61.81	14.31	68.66

Table S4. Effects of promoter-filled operations on absorption flux improvements I_N.

C_{in} (%)	$q_b \times 10^6$ (m³ s⁻¹)	Promoter-filled channel configurations with descending hydraulic diameters				
		Empty channel	Circle type		Diamond type	
		$J_{empty} \times 10^4$ (mol m⁻² s⁻¹)	$J_{circle} \times 10^4$ (mol m⁻² s⁻¹)	I_N (%)	$J_{diamond} \times 10^4$ (mol m⁻² s⁻¹)	I_N (%)
30	2.5	6.70	10.06	50.24	10.53	57.18
	3.33	7.39	10.79	44.43	11.09	50.07
	4.17	7.90	11.22	41.94	11.44	44.71
	5.0	8.08	11.31	39.99	11.50	42.39
35	2.5	7.08	11.37	60.54	12.02	69.70
	3.33	7.68	11.90	54.91	12.48	62.37
	4.17	8.06	12.21	51.53	12.71	57.81
	5.0	8.23	12.28	49.12	12.89	56.59
40	2.5	7.53	12.55	66.59	13.50	79.12
	3.33	8.00	13.16	64.52	13.84	72.96
	4.17	8.33	13.61	63.49	14.03	68.52
	5.0	8.48	13.67	61.17	14.12	66.42

Table S5. Theoretical predictions of the absorption flux improvement I_N and further absorption flux enhancement E_P in the module with descending promoter-filled channels.

C_{in} (%)	$q_b \times 10^6$ m³/s	Descending promoter-filled channels					
		Big - circle	Big - diamond	Descending circle-type		Descending diamond-type	
		I_N (%)	I_N (%)	I_N (%)	E_P (%)	I_N (%)	E_P (%)
30	2.5	31.48	38.24	50.24	14.27	57.18	9.61
	3.33	30.95	37.50	44.43	13.06	50.07	9.14
	4.17	25.96	34.11	41.94	12.69	44.71	7.91
	5.0	24.62	32.05	39.99	12.33	42.39	7.82
35	2.5	38.68	50.77	57.60	15.77	69.70	12.55
	3.33	35.39	45.32	54.91	14.42	62.37	11.74
	4.17	33.18	41.76	51.53	13.78	57.81	11.32
	5.0	31.66	40.99	49.12	13.27	56.59	11.07
40	2.5	38.80	56.14	66.59	20.02	79.12	14.72

	3.33	38.08	51.00	64.52	19.15	72.96	14.54
	4.17	37.79	50.21	63.49	18.65	68.52	12.19
	5.0	37.59	49.48	61.17	17.13	66.42	11.33
